# Comparative Study on Estimation Methods of Dynamic Resistance Using Dynamic Cone Penetrometer

**DOI:** 10.3390/s21093085

**Published:** 2021-04-28

**Authors:** Sang Yeob Kim, Jong-Sub Lee, Dong-Ju Kim, Yong-Hoon Byun

**Affiliations:** 1 Department of Civil and Environmental Engineering, University of Illinois at Urbana-Champaign, 205 North Mathews Avenue, Urbana, IL 61801, USA; s3778@illinois.edu; 2 School of Civil, Environmental and Architectural Engineering, Korea University, 145 Anam-ro, Seongbuk-gu, Seoul 02841, Korea; jongsub@korea.ac.kr (J.-S.L.); kyrix1028@korea.ac.kr (D.-J.K.); 3 School of Agricultural Civil & Bio-Industrial Engineering, Kyungpook National University, 80 Daehak-ro, Buk-gu, Daegu 41566, Korea

**Keywords:** *DCP*, dynamic penetration, dynamic resistance, integration method, transferred energy

## Abstract

Dynamic resistance, which can be used to express strength in the unit of stress and improve the reliability of the dynamic cone penetration test (*DCPT*), has been estimated by numerous methods. This study aims to compare different dynamic resistance estimation methods by using an instrumented dynamic cone penetrometer (*IDCP*). DCPTs are conducted using a standard dynamic cone penetrometer (*DCP*) and *IDCP* in the laboratory and field. Dynamic responses are obtained from the strain gauges and an accelerometer installed at the cone tip of the *IDCP*. The test results show that dynamic resistance is more efficient in distinguishing profiles than the dynamic cone penetration index. Among the methods to estimate the dynamic resistance at the cone tip, the force-velocity integration method and force integration method are more related to the conventional dynamic resistance considering the potential energy of the hammer than the force squared integration method. Additionally, the dynamic resistance estimated for a longer time period is more reliable, particularly for small driving rod lengths. Regarding the limitation of the dynamic response from an accelerometer in a previous study, the force-based dynamic resistance estimated for a longer time period can be used as the most reliable approach for further soil strength characterization.

## 1. Introduction

For subgrade characterization, numerous portable in situ devices have been used to assess the mechanical properties [1,2,3]. Among the portable in situ devices, the dynamic cone penetrometer (*DCP*) has been conventionally adopted in road and railway substructures owing to its rapid and simple procedure [4,5]. The dynamic cone penetration index (*DCPI*), which is a strength profiling index from the *DCP* test, has been widely correlated with several engineering properties such as the California bearing ratio (CBR) and deflection modulus [6,7]. The strength and stiffness indices determined from these correlations have been used for the road pavement design in the transportation engineering field [8,9].

In general, the *DCPI* is measured at each dynamic impact using a free-falling hammer with identical potential energy. However, the transferred energy, which causes the driving force at the cone tip, varies during penetration due to energy loss [10,11]. The energy loss may occur owing to the friction between the hammer and guide, connections of driving rods, and friction between driving rod and soils [2]. When the *DCP* is tilted, the unreliable *DCPI* profile can be obtained during the penetration [11]. Therefore, the profiling results by *DCPI* have a limitation, particularly in reliability, despite the many advantages of using *DCP*. Previous studies examined the factors of energy loss and explored methods to obtain more reliable profiling results [12,13]. Byun and Lee [2] reported that the energy transferred at the cone tip could be evaluated by installing an energy module composed of strain gauges and an accelerometer. On the other hand, the transferred energy during dynamic penetration can be overestimated or underestimated due to the large displacement or limitations of the accelerometer [14].

Several studies have attempted to estimate the dynamic resistance based on the principle of force divided by the cross-sectional area of the penetrometer [15,16]. Lee et al. [17] reported that the dynamic resistance is less affected by the transferred energy and other factors, such as tilting. Kianirad et al. [18] also installed strain gauges at the cone tip to obtain the force signal, and to estimate the dynamic resistance. Kim et al. [19] used both the force and velocity signals because the dynamic resistance can be affected by the penetration rate at the moment of impact. Consequently, there are methods to estimate the dynamic resistance. The *DCP* test can be improved by considering the possible and diverse estimation approach.

This study compares different dynamic resistance estimation methods during *DCP* tests. The *DCP* tests are conducted in the laboratory and field using a standard *DCP* and an instrumented *DCP* (*IDCP*), which is incorporated by strain gauges and an accelerometer. This paper introduces several equations to estimate dynamic resistance using different integration methods and time periods for integration. Subsequently, the estimated dynamic resistances are compared to investigate the effect of each factor, which are assessed by correlation analysis to determine the reliability.

## 2. Dynamic Cone Penetrometers

### 2.1. Standard DCP

The standard *DCP* consists of a hammer, guide, anvil, driving rod, and cone tip [20]. The dynamic impact for the penetration was performed by dropping a 78.5 N hammer from a free-falling height of 575 mm. The impact energy was transferred through the driving rod with a diameter of 16 mm, and the energy transferred at the cone tip with a diameter of 20 mm leads to the cone penetration into the subgrade. For each dynamic impact, the *DCPI* is measured, which is used for continuous subgrade profiling. The *DCPI* can be simply obtained using the following equation:

(1) $$DCPI[mm/blow]=D_{n+1}-D_{n}$$

where *D_n_* is the penetration depth of the *DCP* at a blow count of *n*. The *DCPI* is the only obtainable strength index from the *DCP* test, and it depends on the energy transferred at the cone tip that might reduce the reliability of the *DCP* results.

### 2.2. Instrumented DCP

**Figure 1:**
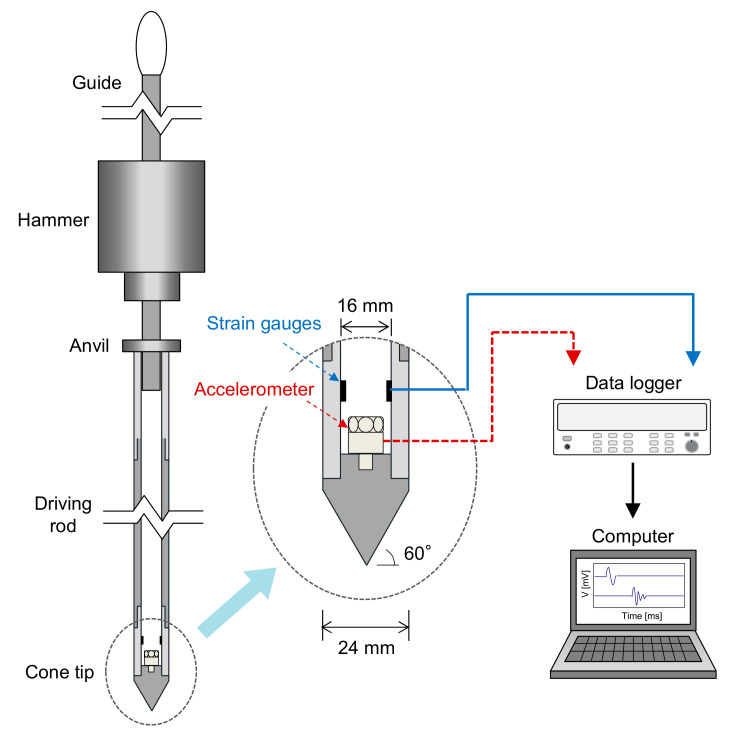
Schematic drawing of instrumented dynamic cone penetrometer with measurement system.

In this study, an *IDCP* was designed to improve the limitations of the *DCP*. The *IDCP* has the same composition as the *DCP*, as shown in Figure 1. However, the diameter of the *IDCP* driving rod was designed to be 24 mm, which is slightly larger than the *DCP*, to secure space for the installation of sensors. Note that a diameter of accelerometer sensor is 10 mm, which is tight to be installed inside the driving rod of *DCP*. Considering the diameter of accelerometer, the thickness of *IDCP* driving rod is designed to be 4 mm, so that the inner diameter of 16 mm secures the space of 6 mm for the installation of accelerometer, as shown in Figure 1. Figure 1 shows that the strain gauges and an accelerometer are installed close to the cone tip. Four strain gauges with a 120 Ω resistance are configured with a full-bridge circuit used widely for temperature compensation and to minimize eccentricity [21]. An accelerometer with a measurement range of 10,000 g was installed at the identical location of the strain gauges. Note that the strain gauges and an accelerometer were calibrated to convert the electrical signals to a force and acceleration, respectively. Finally, all the electrical signal data were visualized and stored on a computer through a data logger with a 96 kHz sampling rate.

## 3. Experimental Study

### 3.1. Cone Resistance Profiles

**Figure 2:**
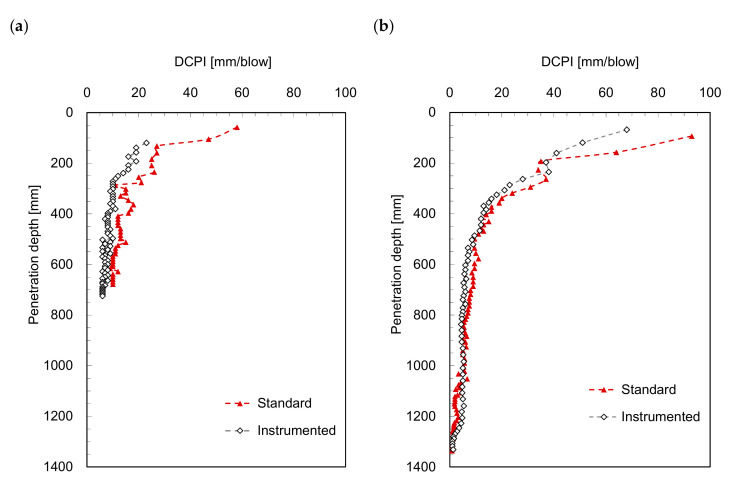
Profiles of dynamic cone penetration index obtained in: (**a**) laboratory model test; (**b**) field test.

Dynamic penetration tests were conducted using a standard *DCP* and an *IDCP* in the laboratory and field. For the laboratory test, weathered soils commonly found in South Korea were sampled for the preparation of specimen in a chamber. The index properties of granular weathered soils were obtained from sieve analysis, and the mean particle size (D_50_), coefficient of curvature (C_c_), and coefficient of uniformity (C_u_) were 0.57 mm, 1.2, and 11.1, respectively. Furthermore, the main components of weathered soils were quartz (32.8%) with low clay minerals. The weathered soils were dried in an oven before the preparation to minimize electrostatic force effect. Finally, the weathered soils were prepared in a chamber with dynamic compaction for five layers with 56 blows using a 44.5 N rammer. Note that the amount of soils for each layer was identical to maintain a target relative density of 97%. Thereafter, both *DCP* and *IDCP* were penetrated into the prepared weathered soils while considering the boundary effect (refer to Lee et al. [17]). For the field test, the *DCP* and *IDCP* were also applied within a 50 cm distance between the two holes. The *DCPI*, one of the resistance profiles obtained from the dynamic penetration test, was determined from Equation (1) and is plotted in Figure 2. The *DCPI* profiles of both *DCP* and *IDCP* show similar trends with a slight difference, because the dimensions of the cone tip and driving rod for the *IDCP* are different than those for the *DCP*. The *DCPI* obtained from the *DCP* varies more sensitively along the penetration depth than that obtained from the *IDCP*. Figure 2 shows that the *DCPI* continuously decreases with an increase in the penetration depth because of the increasing confining stress [22]. Notably, a lower *DCPI* denotes higher soil strength.

**Figure 3:**
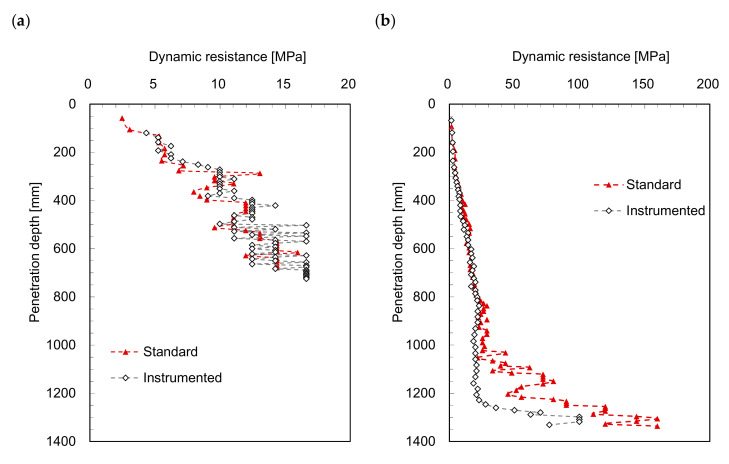
Profiles of dynamic resistances obtained in: (**a**) laboratory model test; (**b**) field test.

For a more direct indication of soil strength, the dynamic resistance (*DR*) in the unit of stress was calculated as follows:

(2) $$DR_{s(or)i}=\frac{1}{A}\times \frac{PE}{\Delta d}=\frac{1}{A}\times \frac{mgh}{\Delta d}$$

where *A*, *PE*, and ∆*d* denote the cross-sectional area of the cone tip, potential energy of the hammer, and penetration distance per blow, respectively. Previous studies initially used potential energy by multiplying the hammer weight (*mg*) with its falling height (*h*) [15,23]. The dynamic resistances (*DR_s_* and *DR_i_*) calculated by Equation (2) from both the *DCP* and *IDCP* tests in this study are plotted in Figure 3. Compared with the *DCPI* profiles in Figure 2, the dynamic resistances from *DCP* and *IDCP* show similar trends with a smaller difference. Notably, the difference in cone tip diameter between the *DCP* and *IDCP* was considered for dynamic resistance estimation. However, the different rod diameters of the *DCP* and *IDCP* may lead to a difference in the dynamic resistance at the same depth, and the discrepancy between the respective dynamic resistances at greater depths is significant. Furthermore, the dynamic resistance continuously increases with increase in penetration depth owing to the confining stress effect, similar to that observed in the *DCPI* results. Particularly, for higher strengths at larger penetration depths, the dynamic resistance is more efficient and clearer in distinguishing the profiles than *DCPI*.

### 3.2. Dynamic Resistance at Cone Tip

**Figure 4:**
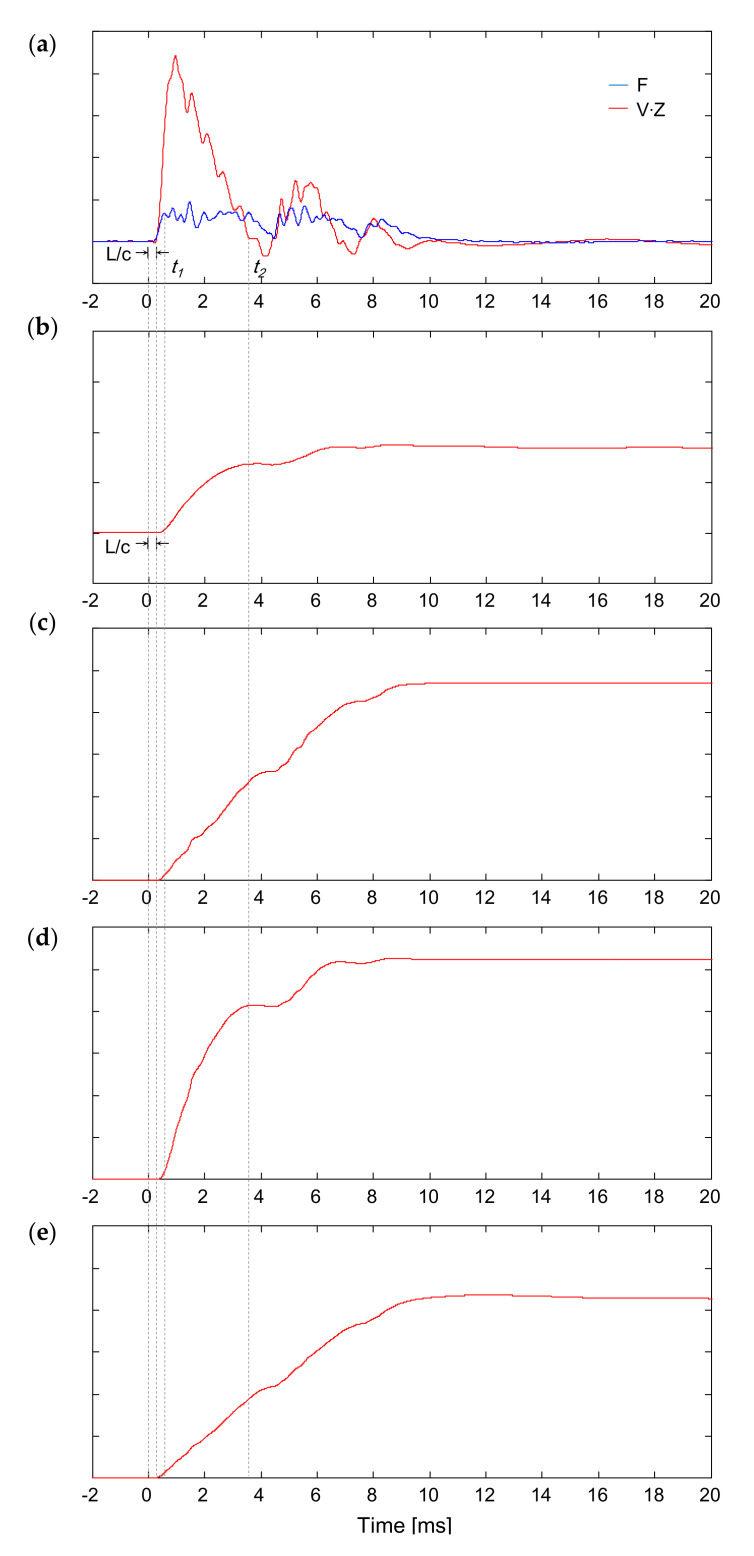
Typical dynamic responses obtained at the cone tip of *IDCP*: (**a**) force-velocity; (**b**) displacement; (**c**) force square integration; (**d**) transferred energy; (**e**) force integration.

To record the dynamic responses and evaluate the transferred energy at the cone tip, strain gauges and an accelerometer were installed at the cone tip (see Figure 1). Using the energy transferred at the cone tip may improve the reliability of the estimation and characterization of cone resistance along the penetration depth. Previous studies suggested various types of dynamic cone resistances based on the transferred energy [17,19,24]. Considering that the force squared integration method (*F*^2^ method) and force–velocity integration method (*F*–*V* method) are widely used for evaluating the transferred energy at the rod head, three different types of dynamic resistances can be calculated as follows:

(3) $$DR_{i1}=\frac{1}{A}\times \frac{1}{\int_{0}^{t_{1}}V\;dt}\times \left(\frac{c}{AE}\int_{0}^{t_{1}}F^{2}\;dt\right)$$

(4) $$DR_{i2}=\frac{1}{A}\times \frac{\int_{0}^{t_{1}}F\times V\;dt}{\int_{0}^{t_{1}}V\;dt}$$

(5) $$DR_{i3}=\frac{1}{A}\times \frac{\int_{0}^{t_{2}}F\times V\;dt}{\int_{0}^{t_{2}}V\;dt}$$

where *c* and *E* denote the wave velocity through the steel rod and Young’s modulus of the rod, respectively. The term t denotes the time elapsed at the cone tip and 0 indicates the initial rising time at the rod head. The terms *t*_1_ and *t*_2_ denote the times corresponding to *L*/*c* and the first zero-velocity at the cone tip, respectively. The terms *L*, *F*, and *V* denote the rod length, force measured from the strain gauges, and particle velocity estimated from the accelerometer, respectively. The particle velocity, which can be calculated by integrating the acceleration, is multiplied by the impedance of the material to be compared with the force signal. The typical dynamic responses obtained from Equations (3)–(5) are plotted in Figure 4. Figure 4a shows the force and velocity signals from the strain gauges and accelerometer, respectively. The first peak velocity was significantly higher than the peak force because the sensors were installed at the cone tip. Additionally, the initial rising time of both the force and velocity signals is the time corresponding to *L*/*c* at the rod head, and it is equal to the travel time of the compressional waves propagated from the rod head to cone tip. Figure 4b shows the variation in displacement calculated by the integration of velocity, which can be substituted into the denominators in Equations (3)–(5). Notably, the displacement in Equations (3)–(5) can be calculated using the signal recorded at the cone tip, while ∆*d* in Equation (2) is obtained by directly measuring the penetration depth.

Recently, a force-based dynamic resistance has also been suggested by several researchers [18,19,25]. By adopting the same interval of integration as that used in Equations (3)–(5), the dynamic resistances based on the force integration (*F* method) can be expressed as follows:

(6) $$DR_{i4}=\frac{1}{A}\times \frac{\int_{0}^{t_{1}}F\;dt}{\Delta t}$$

(7) $$DR_{i5}=\frac{1}{A}\times \frac{\int_{0}^{t_{2}}F\;dt}{\Delta t}$$

where Δ*t* denotes the duration between zero and *t_1_* or *t_2_*. The typical variation in force integration calculated by Equations (6) and (7) is plotted in Figure 4e. Using the *F*^2^, *F*, and *F*–*V* methods, five types of dynamic resistances can be obtained by two different integration intervals. Notably, different time periods for integration were adopted in previous studies [17,25].

**Figure 5:**
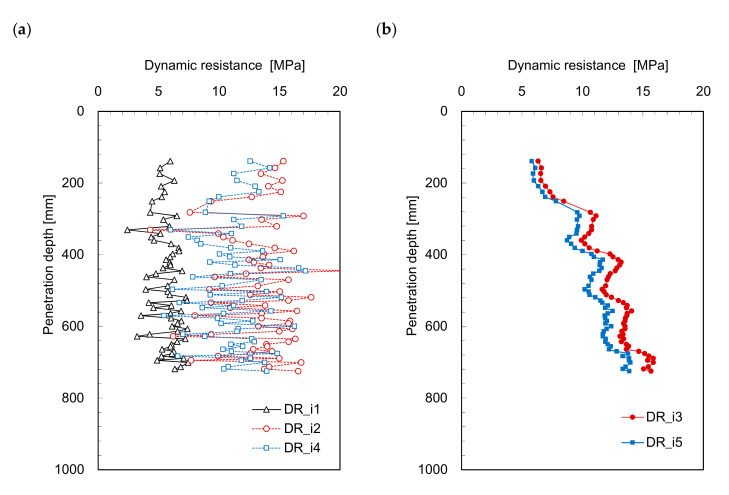
Profiles of dynamic resistances in laboratory model estimated at the different durations from zero to: (**a**) *t*_1_; (**b**) *t*_2_.

**Figure 6:**
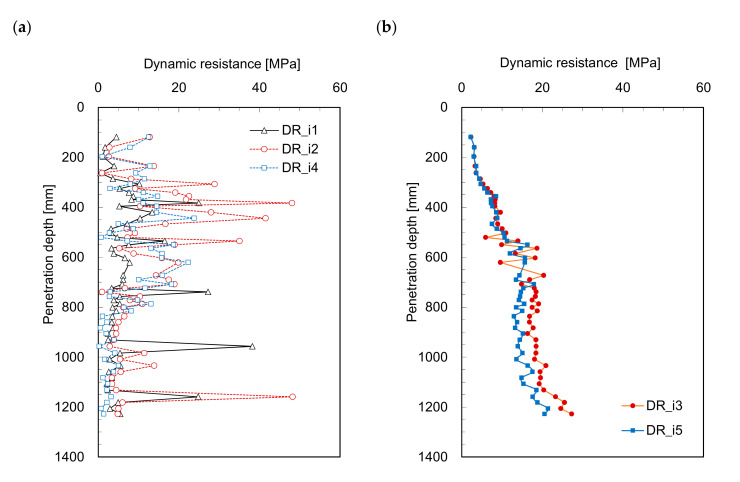
Profiles of dynamic resistances in field estimated at the different durations from zero to: (**a**) *t*_1_; (**b**) *t*_2_.

**Figure 7:**
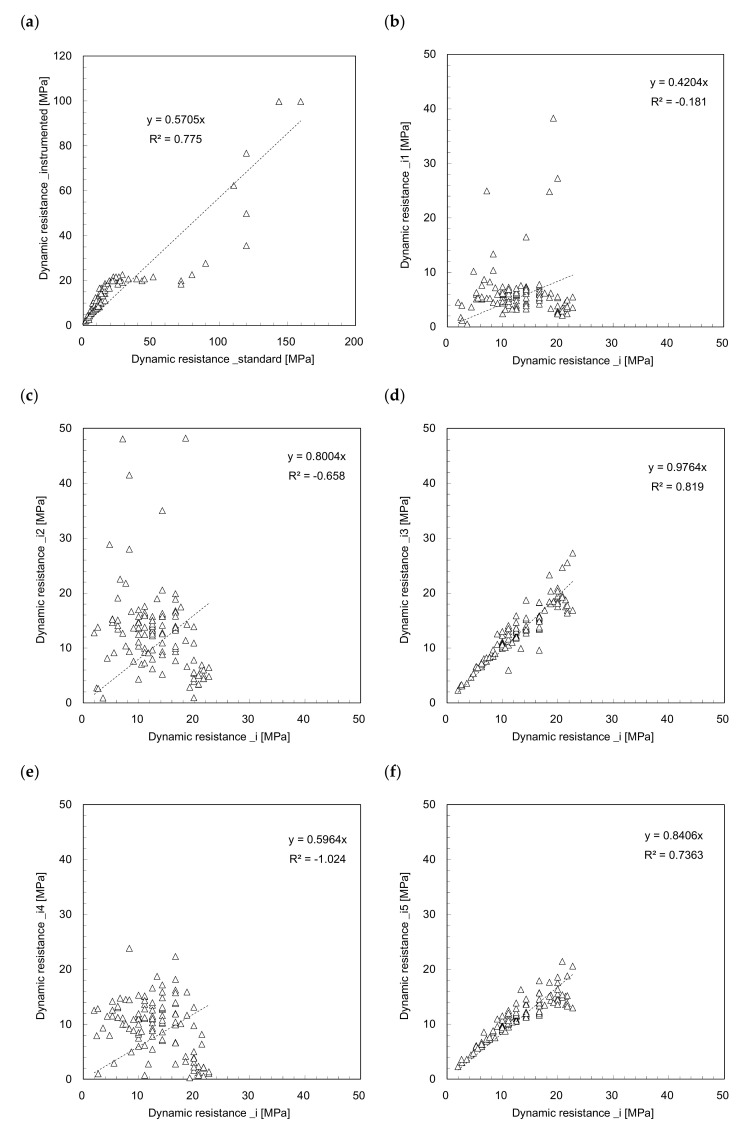
Comparison of dynamic resistances between: (**a**) *DR_s_‒DR_i_*; (**b**) *DR_i_‒DR_i_*_1_; (**c**) *DR_i_‒DR_i_*_2_; (**d**) *DR_i_‒DR_i_*_3_; (**e**) *DR_i_‒DR_i_*_4_; (**f**) *DR_i_‒DR_i_*_5_.

The dynamic resistance profiles estimated from Equations (3)–(7) are plotted in Figure 5 and Figure 6. Figure 5a and Figure 6a show the dynamic resistance profiles for the time period from 0 to *t*_1_, and the dynamic resistances significantly fluctuate along the penetration depth. Contrarily, the dynamic resistances (*DR_i_*_3_ and *DR_i_*_5_) for the time period 0 to *t*_2_ (see Figure 5b and Figure 6b), increase with the penetration depth; this is similar to the trends of the dynamic resistance profiles (see Figure 3). The dynamic resistance generally increases with depth owing to the confining stress (see Figure 3). Additionally, *DR_i_*_3_ and *DR_i_*_5_ present notably similar trends with a very slight difference. All the dynamic resistances obtained from the *IDCP* are plotted in Figure 7 to compare the relationships among the estimated values. Figure 7a shows the dynamic resistances obtained using Equation (2) from the standard *DCP* and *IDCP*, respectively, and most dynamic resistances from the *IDCP* are higher than those from the standard *DCP*. Considering the difference in tip shape between the *IDCP* and *DCP*, the higher friction along the rod of the *IDCP* may lead to overestimation of the dynamic cone resistance (*DR_i_*). Among the relationships between the dynamic resistance (*DR_i_*) and those (*DR_i_*_1_ to *DR_i_*_5_) from Equations (3)–(7), the relationships of *DR_i_*–*DR_i_*_3_ and *DR_i_*–*DR_i_*_5_ in Figure 7d,f show a linear trend with a relatively higher coefficient of determinant. Considering that *DR_i_*_3_ and *DR_i_*_5_ were obtained from a longer interval compared with *DR_i_*_1_, *DR_i_*_2_, and *DR_i_*_4_, it was found that *DR_i_* is more related to the dynamic resistance estimated for longer time periods.

### 3.3. Comparison

**Figure 8:**
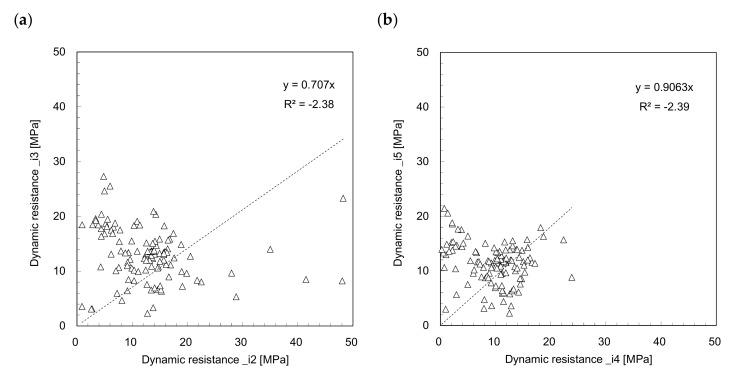
Comparison of dynamic resistances between: (**a**) *DR_i_*_2_*‒DR_i_*_3_; (**b**) *DR_i_*_4_*‒DR_i_*_5_.

**Figure 9:**
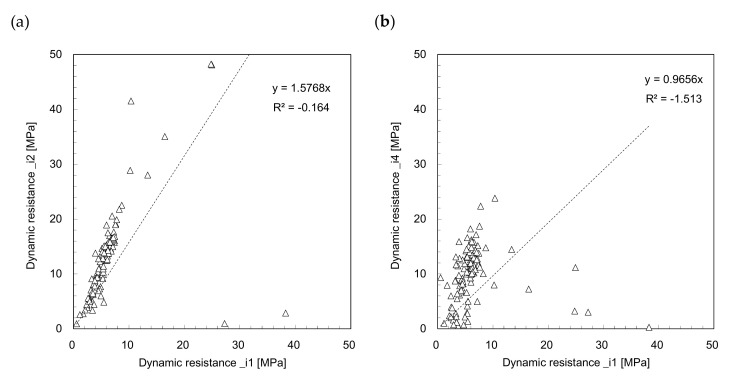
Comparison of dynamic resistances between: (**a**) *DR_i_*_1_*‒DR_i_*_2_; (**b**) *DR_i_*_1_*‒DR_i_*_4_.

**Figure 10:**
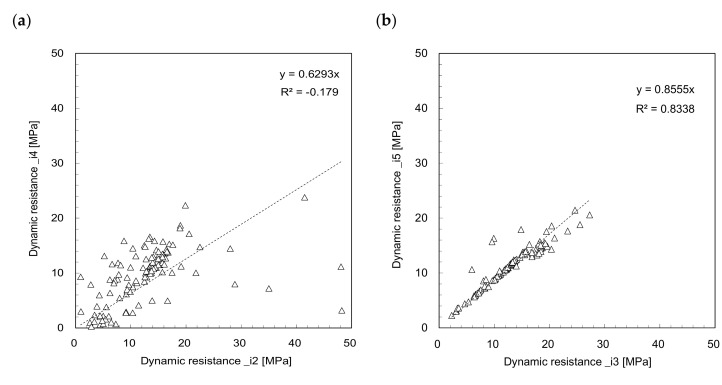
Comparison of dynamic resistances between: (**a**) *DR_i_*_2_*‒DR_i_*_4_; (**b**) *DR_i_*_3_*‒DR_i_*_5_.

In this study, the type of dynamic resistance determined using the sensors can be allocated to three integration methods with two different intervals. To investigate the effect of integration interval on dynamic resistance estimation, the relationships of *DR_i_*_2_–*DR_i_*_3_ and *DR_i_*_4_–*DR_i_*_5_ are plotted in Figure 8. Figure 8 shows that the dynamic resistances are scattered even for the same integration method, and that they have an insignificant relationship when estimated at different intervals. For the same interval of 0 to *t*_1_, the relationships of *DR_i_*_1_–*DR_i_*_2_ and *DR_i_*_1_–*DR_i_*_4_ are plotted in Figure 9 to compare the *F*^2^ method with the *F*–*V* and *F* methods. Figure 9 shows that most *DR_i_*_2_ and *DR_i_*_4_ are greater than *DR_i_*_1_. The relationship between the dynamic resistances estimated from the *F*^2^ and *F*–*V* methods presents a linear trend, as shown in Figure 9a. Figure 9b shows a linear relationship between the dynamic resistances estimated from the F^2^ and F methods. The linear trend for *F*^2^ and *F*–*V* methods has higher coefficient of determination than the trend for F^2^ and F methods. To compare the dynamic resistances estimated from the *F*–*V* method with those estimated from the F method for the same interval, the relationships of *DR_i_*_2_–*DR_i_*_4_ and *DR_i_*_3_–*DR_i_*_5_ are plotted in Figure 10. The relationship estimated for the interval of 0 to *t*_2_ shows a higher coefficient of determinant than that estimated for the interval of 0 to *t*_1_. The relationship between *DR_i_*_3_ and *DR_i_*_5_ estimated for the interval of 0 to *t*_2_ is the most reliable, as mentioned previously. Considering that the driving rod length for the *DCP* is small and that the interval of 0 to *t*_1_ is extremely short, the longer interval of 0 to *t*_2_ is more reasonable for estimating the dynamic resistance using the *IDCP*. Lee et al. [17] also reported that the dynamic cone resistance estimated from the *F* method for the interval of 0 to *t*_2_ was strongly correlated with strength parameters, such as *DCPI*, internal friction angles, and CBR values. According to Kim et al. [26], in the view of resonant frequency, the dynamic responses obtained from strain gauges are more reliable than those obtained from accelerometers. Therefore, the effect of integration interval on dynamic resistance estimation is significant, regardless of the integration methods, and the force-based dynamic resistance estimated for longer intervals would be a promising approach for soil strength characterization. Considering that previous studies simulated the characteristics and behavior of granular materials under similar penetration condition [27,28], further study on the simulation of dynamic penetration in the granular material may be meaningful to better understand the dynamic resistances estimated by using *IDCP*.

## 4. Summary and Conclusions

This study mainly considered an *IDCP* to compare the dynamic resistances estimated by different methods. The standard *DCP* and an *IDCP* were used for laboratory and field tests. Strain gauges and an accelerometer were installed at the cone tip of the *IDCP*. The *DCPI* and dynamic resistance, considering the potential energy from the *DCP* and *IDCP* tests, were estimated. Thereafter, the dynamic resistances estimated by different integration methods and time periods using dynamic responses at the cone tip were compared, and the most important findings are as follows:

- Dynamic cone resistances, considering the potential energy from the *DCP* and *IDCP*, showed a similar trend with a slight difference. Particularly, the dynamic resistance continuously increased with increase in penetration depth owing to the confining stress effect, whereas the *DCPI* decreased. Thus, for higher strength at deeper penetration depth, dynamic resistance is more efficient in distinguishing the profiles than *DCPI*;
- For the relationships between dynamic resistances determined using potential energy and dynamic response at the cone tip, the *F*–*V* and *F* methods for longer time periods present a linear trend with a high coefficient of determination;
- Among the dynamic resistances estimated by using the dynamic responses at the cone tip, the relationships between short and long time periods with the same integration methods showed an insignificant trend. Meanwhile, the relationship between the *F*^2^ and *F*–*V* methods was more reliable than that between the *F*^2^ and *F* methods for a short time period. However, for long time periods, the relationship between the *F*–*V* and *F* methods was the most reliable than the other relationships;
- Consequently, the *F*–*V* and *F* methods for long time periods are efficient for obtaining reliable estimations. Furthermore, considering the limitation of accelerometers, the force-based dynamic resistance for long time periods could be a promising approach for soil characterization.

## Data Availability

The data presented in this study are available on request from the corresponding author. The data are not publicly available due to ongoing research project.
